# Multiple spermatophore transfer during prolonged mating in the apterous neotropical cricket *Unacla meridionalis* (Desutter-Grandcolas 1994) (Orthoptera: Grylloidea: Phalangopsidae)

**DOI:** 10.1007/s13744-026-01449-9

**Published:** 2026-11-13

**Authors:** Leanna Camila Macarini, Jessica Ricci de Lima, Marcos Fianco, Edison Zefa, João Pedro Maruzka Roncaglio, Vitória Garcia de Camargo, Neucir Szinwelski

**Affiliations:** 1 Universidade Estadual do Oeste do Paraná, Cascavel, PR Brazil; 2 Universidade Federal do Paraná, Curitiba, PR Brazil; 3 Universidade Federal de Pelotas, Pelotas, RS Brazil

**Keywords:** Insect, Ensifera, Courtship, Copulation, Nuptial gift, Atlantic Forest

## Abstract

Phalangopsidae comprises over 1,136 species of crickets, but reproductive behavior has been documented for only a few species. Here, we describe the mating behavior of *Unacla meridionalis* (Desutter-Grandcolas 1994), an apterous, nocturnal cricket endemic to the Brazilian Atlantic Forest. We analyzed 15 mating trials conducted under controlled laboratory conditions, including courtship, copulation, and post-copulation behaviors. During courtship, a male performs dorsoventral vibrations of the body and touching with the antennae, while a female palpates the abdominal tergites of the male; spermatophores are transferred during subsequent copulations; after the mating pair separated, females consumed the spermatophores in 87% of the encounters, which contrasts with the typical spermatophore consumption by males in other phalangopsid species and suggests that the spermatophore may represent a post-nuptial gift. The total mating event lasted approximately 2 h, representing a considerably long duration. These findings expand our understanding of the diversity of reproductive strategies within Phalangopsidae and underscore the evolutionary significance of extended mating and paternal investment in apterous crickets.

## Introduction

Reproductive behavior in insects is particularly diverse and complex, involving a suite of courtship, copulation, and post-copulation interactions shaped by sexual selection (Andersson 1994; Adams and Tariq 2024). In these contexts, females typically choose mates due to their higher energetic investment in offspring, using male signals to assess genetic quality and ensure reproductive success (Kelly 2018).

Acoustic communication in crickets (Orthoptera: Grylloidea) is one of the most studied aspects of mating behavior, with males typically producing calling and courtship songs through tegminal stridulation to attract and stimulate females (Alexander 1962; Mowles 2014). However, other forms of communication, such as substrate-borne vibrational signaling (Stritih-Peljhan and Virant-Doberlet 2021) and chemical signaling (Stritih and Žunič Kosi 2017) can be used by crickets, especially in apterous species that lack the tegminal apparatus required for sound production (Desutter-Grandcolas 1995; Stritih-Peljhan and Virant-Doberlet 2021).

Phalangopsidae comprises 1.136 valid species (Cigliano et al. 2026), encompassing apterous, micropterous, and macropterous crickets with diverse ecological habits (Desutter-Grandcolas 1992). Despite this remarkable species richness, reproductive behavior has been described for only about 1% of the species (Macarini et al. 2023), leaving a substantial gap in our understanding of behavioral diversity. Among the few behavioral descriptions available, notable variation has been documented in courtship signals, copulatory mechanics, and post-copulatory investment, suggesting that Phalangopsidae harbors a yet-unexplored diversity of mating strategies (Desutter-Grandcolas 1995; Zefa et al. 2008; Fianco et al. 2018; Macarini et al. 2023).

*Unacla meridionalis* (Desutter-Grandcolas 1994) is a nocturnal, completely apterous cricket endemic to the Atlantic Forest of southern and southeastern Brazil (Desutter-Grandcolas 1994; Mews et al. 2008). As a wingless species with no described glandular structures, its mating communication is expected to rely on alternative sensory modalities such as vibrational signals, chemoreception, and tactile cues, making it a particularly interesting model for understanding reproductive behavior in the absence of acoustic signaling.

Here, we provide a comprehensive description of the complete mating repertoire of the cricket *U. meridionalis*, including courtship, copulation, and post-copulation stages. Our findings contribute to narrowing the knowledge gap on the reproductive biology of phalangopsid crickets and provide new evidence for the evolution of extended mating and nuptial investment strategies in apterous crickets.

## Material and Methods

### Target Species

**Fig. 1:**
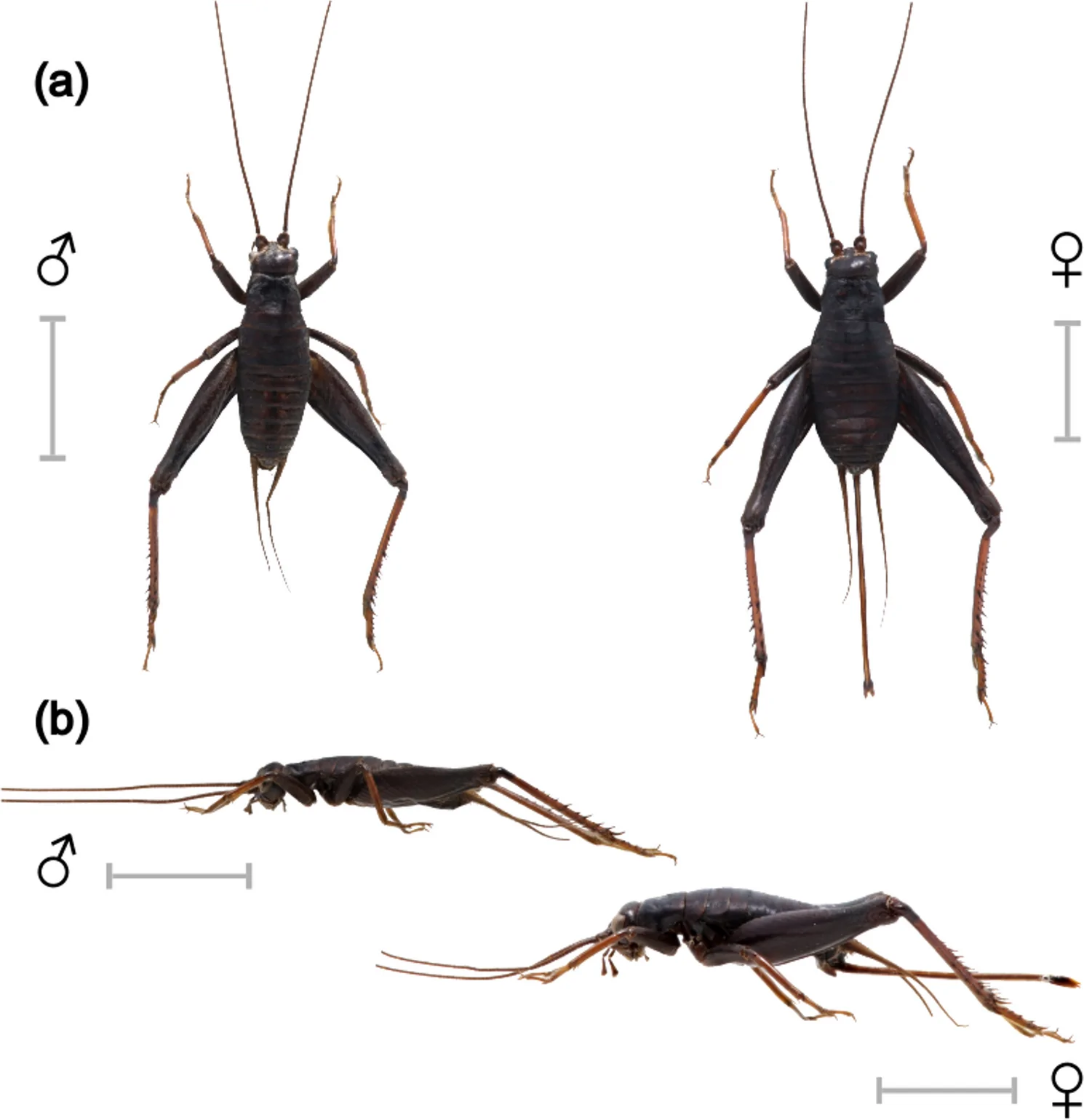
Male (♂) and female (♀) of *Unacla meridionalis*. (**a**) Dorsal and (**b**) lateral views, post-fixation, detailing the external morphology. Grey scale bars represent 1 cm

*Unacla* (Campos and Souza-Dias 2025) crickets exhibit a distinctive morphology compared to other Phalangopsidae. Their dorsoventrally flattened body is likely an adaptation for occupying sheltered microhabitats, such as spaces beneath bark and narrow tree crevices, during the day (Desutter-Grandcolas 1994). *Unacla meridionalis* is a nocturnal cricket, completely wingless with no documented associated metanotal glandular structures. The males have a glossy dark brown body with complex, large genitalia with ventral projections; females are slightly larger than males, exhibit the same body coloration, and possess a long ovipositor (Desutter-Grandcolas 1994) (Fig. 1).

### Sampling and Laboratory Rearing

Individuals of *U. meridionalis* were collected at night from logs, branches of living or dead trees, shrubs, and leaf litter in Iguaçu National Park, Foz do Iguaçu – PR (25° 22′ 24″ S – 54° 2′ 33″ W), between March and September 2016. Upon collection, the specimens were transported to the Agricultural Biotechnology Laboratory of the Universidade Estadual do Oeste do Paraná (Unioeste), Cascavel, PR, Brazil, where they were identified and kept separately in transparent plastic containers (7 cm high × 11 cm diameter – 500 mL), with openings in the lids for ventilation.

Shelters made from egg cartons and pieces of tree branches were added to each container. All individuals were maintained in a climate-controlled room with 75% relative humidity, a temperature of 23°C, and a 12-h light–dark photoperiod. Food (commercial fish flakes) and water were provided ad libitum. The crickets were kept in these conditions for 7 days for acclimatization before the start of the experiments.

### Reproductive Behavior

For the reproductive behavior experiments, a total of 170 adult crickets (85 males and 85 females) were randomly paired to form 85 unique couples, with each specimen tested in a single trial. Because individuals were collected as adults directly from their natural habitat, the prior mating status of the males and females was unknown, and they were assumed to be non-virgin.

Firstly, we performed five pilot encounters during the daytime period that resulted in no reproductive behavior. Based on these preliminary trials, all subsequent recordings were performed at night (7:00 pm – 3:00 am), when individuals were active. Thus, all 85 independent mating trials were conducted under nighttime conditions. Trials in which no courtship behavior was observed within 15 min were terminated. As a result, 15 trials culminated in complete copulations and were included in the behavioral analyses.

At the beginning of each trial, a male and a female were placed together in an experimental arena consisting of a rectangular glass container (20 cm long × 15 cm wide × 15 cm high) lined with filter paper, which was replaced after each encounter. Individuals were initially placed on opposite sides of the arena and covered with plastic cups for a two-minute acclimatization period. After simultaneous removal of the cups, interactions were recorded using a Canon camera (PowerShot SX210 IS, 14.1 MP). The video recordings were transferred to a computer and analyzed using standard media playback software. Behavioral acts and stage durations were cataloged by an initial observer and subsequently verified through independent examination by a second observer to ensure consistent classification across all trials. The courtship, copulation, and post-copulation stages were defined, and the duration of each stage was presented as mean ± standard deviation.

## Results

For *Unacla meridionalis*, all mating events, including courtship, copulation, and post-copulation, lasted for 2 ± 0.7 h (n = 15). Stage durations, and frequencies of occurrence of behavioral acts comprising the mating sequence are summarized in Table 1.

**Table 1:** Latencies, stage durations, and occurrence frequencies of the behavioral acts comprising the mating sequence in Unacla meridionalis under laboratory conditions (n = 15 pairs)

*Mating behavior*	*Duration (min)*	*n*	*Occurrence (%)*	*Description*
Courtship				
Initial immobility	10.87 ± 10.28	15	100.0	Period of immobility following pair introduction into the arena
Substrate exploration	1.31 ± 1.52	15	100.0	Active walking accompanied by antennal and palpal palpation of the substrate
Dorsoventral vibration	0.86 ± 0.54	10	66.7	Vibrations of the male body upon perception of the female
Antennal contact	0.30 ± 0.20	12	80.0	Mutual antennation marking the initial physical encounter between the pair
Tergite palpation	1.58 ± 0.88	15	100.0	Female palpation of male 5th–7th abdominal tergites with maxillary and labial palps
Copulation				
Spermatophore exposure	2.00 ± 0.73	15	100.0	Male positioning beneath the female and phallic complex eversion
Genital engagement	6.14 ± 2.85	15	100.0	Terminal back-and-forth attachment movements
Copulatory phase	34.95 ± 19.50	15	100.0	Total duration of genital attachment with spermatophore coupled to the female
Male spermatophore removal	0.67 ± 0.38	15	100.0	Male scraping his abdomen against the substrate to detach remnant structures
Repeated transfers	-	13	86.7	Multiple consecutive copulatory cycles occurring within a single mating event
Post-copulation				
Post-copulatory phase	3.39 ± 2.33	15	100.0	Total elapsed duration of post-copulatory interactions
Female spermatophore removal	-	15	100.0	Female dismounting and bending her abdomen ventrally to extract the spermatophore
Female ingestion	-	13	86.7	Female ingestion of spermatophores functioning as nuptial gifts following repeated transfers
Male ingestion	-	2	13.3	Male ingestion of spermatophores detached and discarded by the female
Complete mating duration				
Complete mating duration	119.13 ± 42.53	15	100.0	Total time elapsed until the completion of all mating interactions

Values are expressed as mean ± standard deviation

Repeated transfers denote the mean number of consecutive transfer cycles per mating event (4 ± 2) rather than duration. Specific durations for individual post-copulatory sub-behaviors are not available as they were quantified as a single aggregate block (post-copulatory phase) across replicates, rather than as isolated continuous metrics. Frequencies and sample sizes (n = 15) refer to pairs that are engaged in complete mating sequences. The overall mating success across all tested pairs in the experimental trials was 17.6% (15 out of 85 pairs)

### Courtship

Males and females of *U. meridionalis* remained motionless for approximately 10.9 ± 10.3 min (n = 15), after release into the arena. After this period, the couples slowly walked around the arena, antennating and palpating the substrate with their maxillary palps (1.3 ± 1.5 min).

**Fig. 2:**
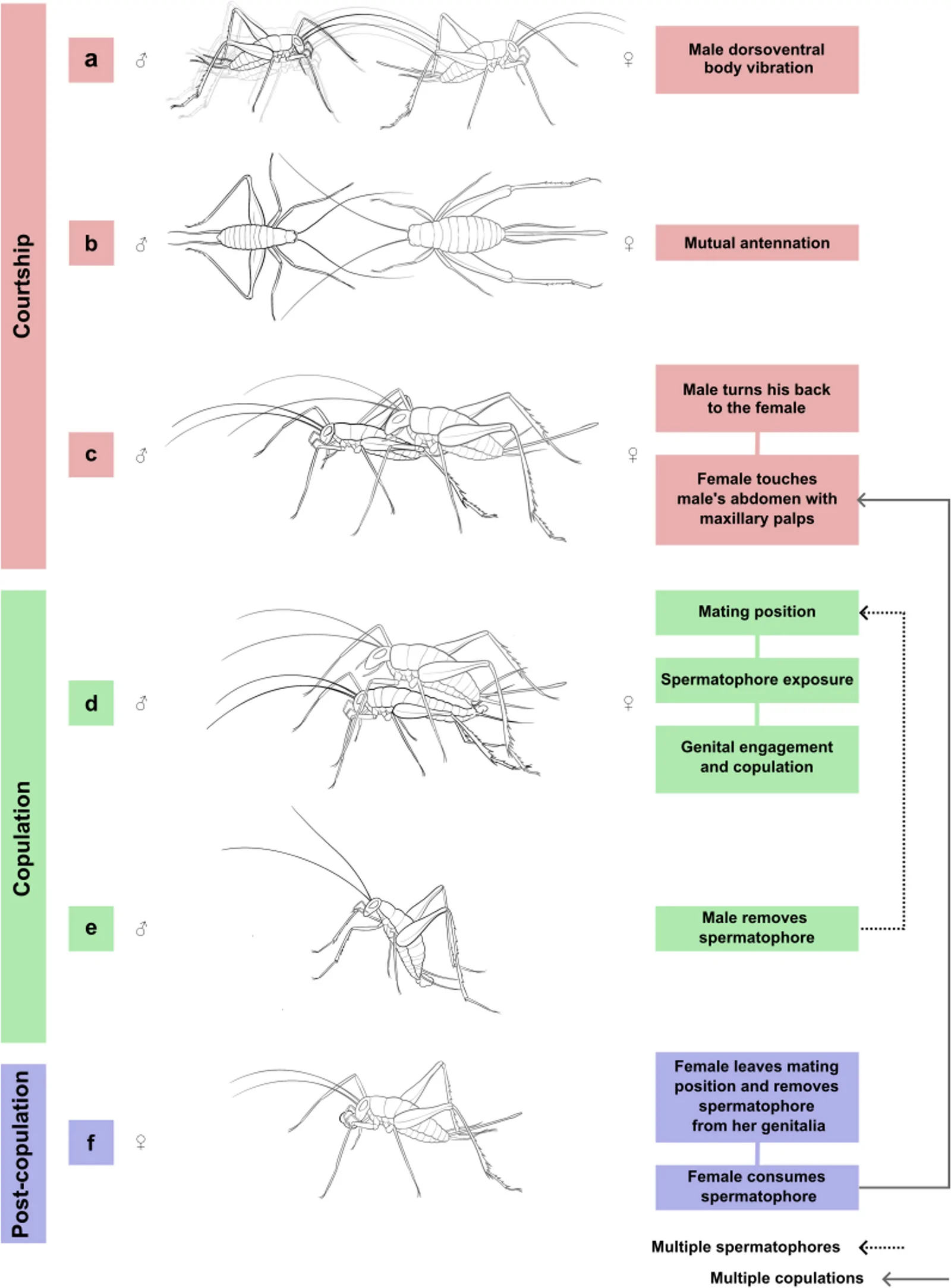
Schematic sequence of the reproductive behavior of *Unacla meridionalis*. Pre-copulation: male performing (**a**) dorsoventral vibration, (**b**) antennation, (**c**) female palpating male's abdomen. Copulation: (**d**) positioning, spermatophore production, and couple mating; (**e**) male removing the spermatophore; post-copulation: (**f**) female consuming the spermatophore. Sequential matings: following spermatophore detachment or ingestion, pairs can resume abdominal inspection and mating position

Upon detecting females in the environment, whether by inspecting the substrate where females had previously passed or by touching any part of a female's body with their antennae, males began to exhibit dorsoventral body vibrations (Fig. 2a) for 51.7 ± 32.3 s (n = 10). Consequently, the first physical contact between the pair occurred through antennation (Fig. 2b), which lasted for 17.7 ± 11.8 s (n = 12). In nine instances, males approached females while antennating, after the immobility period. In the remaining cases (n = 6), females approached the males. Overall, the first physical contact between the pairs occurred 13.3 ± 12.5 min after the start of the experiment (i.e., after the removal of the plastic cups), covering the entire period of immobility as well as the time spent performing body vibrations. In four encounters, the males did not perform body vibrations, and in three, antennal contact was not observed between the pairs, with the interaction proceeding directly to the next stage of reproductive behavior.

When couples were close together, males turned their backs to the females (n = 14), who approached the dorsal abdominal region of males, between the fifth and seventh tergites, palpating the area with their maxillary palps (Fig. 2c). The palpating behavior was observed in all encounters, lasting approximately 94.7 ± 52.5 s (n = 15). In one of the encounters, the male did not vibrate his body, there was no antennal contact between the pair, and in this case, the female went straight to the male's abdomen.

### Copulation

Following courtship, males positioned themselves beneath the females and assumed the copulatory position. They then everted their genitalia, producing and exposing the first spermatophore over 120.3 ± 43.7 s (n = 15). Once the spermatophore was exposed, males tilted their terminalia toward the female genitalia, while females lowered their subgenital plate. To achieve genital engagement, males performed back-and-forth movements with their terminal abdominal segments to attach their phallic complex via the pseudepiphallic arms; these back-and-forth movements lasted 6.1 ± 2.8 min. When successfully attached (Fig. 2d), the spermatophore remained attached to the female genitalia for 35 ± 19.5 min (n = 15).

In two encounters, there was only one copulation, and in the others, males exited the copulation position and removed the spermatophore, scraping the abdomen tip against the substrate (Fig. 2e) for 40.3 ± 23 s. During this period, females remained immobile, and immediately after removing the spermatophore, males returned to the copulatory position and the removed spermatophore remained on the substrate. After the couple re-assumed the copulation position, the male produced a new spermatophore, everted his genitalia again, and copulation proceeded as described above. Following the first copulation, an average of 4 ± 2 (n = 13) additional copulations were observed.

### Post-copulation

The entire post-copulatory phase lasted 3.4 ± 2.3 min and was initiated by the female, who dismounted from the male's back to terminate the copulatory position. To remove the transferred spermatophore, the female bent her body ventrally, utilizing her mandibles and maxillary palps to pull the structure directly from her genital papilla. Concurrently, the male did not remain stationary; upon separation, he immediately moved away and engaged in localized grooming, scraping the terminal segments of his abdomen against the substrate. Following these initial actions, both individuals began to move randomly around the arena, screening the substrate with their antennae. Females consumed the spermatophores (Fig. 2f) that were on the substrate in 87% of the encounters (n = 13). However, when females failed to consume the spermatophore, it was subsequently consumed by the male (n = 2).

## Discussion

This study provides the first comprehensive description of the reproductive behavior of the apterous cricket *Unacla meridionalis*, which lacks the acoustic signaling typically central to cricket mating. Although several behavioral components, such as antennation, body vibrations, abdominal palpation, and female-above copulation, occur in other phalangopsids (Zefa et al. 2008; Fianco et al. 2018; Macarini et al. 2023), the repeated spermatophore transfer, predominant consumption by females, and exceptionally long mating duration represent an unprecedented behavioral repertoire for this cricket family.

In crickets, contact pheromones play a central role in species recognition and in the assessment of mate quality (Kortet and Hedrick 2005), and male body vibrations may enhance the release, dispersal, or perception of these chemical cues (Tishechkin 2022) following initial contact with the female. Under this scenario, the dorsoventral vibrations observed in *U. meridionalis* males may function as short-range mechanical stimuli that complement chemical communication. Furthermore, substrate-transmitted mechanical waves are a widely used signaling modality in cricket sexual communication (Stritih-Peljhan and Virant-Doberlet 2021). Similar vibratory behaviors have been reported during reproductive interactions in other Phalangopsidae species, such as *Vanzoliniella sambophilla* (Mello and Reis 1994) and *Adelosgryllus rubricephalus* (Zefa et al. 2008).

In contrast to the horizontal body rocking characteristic of Gryllidae (Adamo and Hoy 1994), phalangopsid males rely on dorsoventral body tremulations. These vertical oscillations generate substrate-borne vibratory waves that maintain high signal fidelity across short distances regardless of substrate properties. In this context, combining chemical and vibrational signals facilitates reliable partner recognition in the structurally complex bark microhabitats occupied by *U. meridionalis*, thereby circumventing the need for elaborate acoustic or visual signaling. Furthermore, in other crickets, such as *Gryllus bimaculatus*, body oscillations serve dual roles by mediating competitive dominance as well as signaling status during courtship (Vedenina and Shestakov 2018). Future comparative trials will clarify whether *U. meridionalis* vibrations function strictly as precopulatory cues or also convey status during agonistic interactions.

Regarding the sensory system for communication, antennae are multifunctional sensory organs that act as both chemoreceptors and mechanoreceptors, enabling individuals to obtain information about their environment, other organisms, and their specificities through direct physical contact (Loudon et al. 2014; Elgar et al. 2018). This information can reveal mate quality and reproductive status (Sakura and Aonuma 2013), including cues associated with age and sexual maturity (Mello 2007; Rodríguez-Muñoz et al. 2019). The antennation observed in *U. meridionalis* is therefore likely to play a central role in partner assessment and the initiation of courtship behavior.

The pheromonal communication is primarily mediated through antennal contact, although the maxillary and labial palps also contribute to the detection and assessment of chemical cues (Hardy and Shaw 1983; Tregenza and Wedell 1997; Iwasaki and Katagiri 2008). In this way, maxillary palps also function as multimodal sensory organs in crickets, participating in both tactile and chemical perception (Klein 1981; Iwasaki and Katagiri 2008; Matsumoto et al. 2015).

In the reproductive encounters observed in *U. meridionalis*, females palpated the male abdominal tergites with their maxillary palps prior to mounting. This behavior may be related to the female's investigation for physical and chemical cues necessary to trigger mounting (Balakrishnan and Pollack 1997). Furthermore, similar behaviors have been reported in other Phalangopsidae species and have been associated with the presence of metanotal or tergal glands that provide nuptial secretions and may reflect male reproductive investment (Fernandes et al. 2016; Macarini et al. 2023).

During mating, the spermatophore may be displayed to the female as a sexual attribute, as observed in *Adelosgryllus rubricephalus* (Zefa et al. 2008). However, in *U. meridionalis*, the spermatophore is externally exposed only during copulation, preventing it from transmitting visual information to females, who are already on top of the males when the spermatophore becomes visible. In contrast, in all previously studied Phalangopsidae species, the spermatophore has consistently been consumed by the male at the end of copulation (Alexander and Otte 1967; Prado 2006; Fianco et al. 2018; Corrêa et al. 2018; Macarini et al. 2023).

However, in *U. meridionalis*, females consumed the spermatophore in 87% of the encounters, suggesting that it may represent a post-nuptial gift, a form of paternal investment also reported in other cricket species and associated with increased female fecundity and egg quality (Simmons 1988; Shaw and Khine 2004; Prokop and Maxwell 2008). In addition, the transfer of multiple spermatophores during a single mating may increase the amount of sperm and seminal substances received by the female. In orthopterans, these seminal compounds, including prostaglandins and proteins, are known to stimulate egg maturation and oviposition (Andrés et al. 2006; Ryan and Sakaluk 2009; Simmons et al. 2013; Bonilla et al. 2015). Whether multiple spermatophore transfer in *U. meridionalis* provides similar physiological benefits remains to be investigated.

One of the most striking findings of this study is the exceptionally long total duration of *U. meridionalis* mating, as the complete mating event, lasted on average almost two hours. This duration ranks among the longest ever documented for Phalangopsidae, being comparable to *Phaeophilacris bredoides* (Orthoptera, Phalangopsidae), whose mating sequence can also last up to two hours (Lunichkin et al. 2016).

The extended mating in *U. meridionalis* appears to be associated with the production of multiple sequential spermatophores (mean 4 ± 2 per event), to maximize sperm transfer and, consequently, fertilization success (Simmons 1988; Worthington and Kelly 2016). This extended copulation may also function as mate guarding, reducing the likelihood of females remating with rival males during the prolonged coupling period (Sillén-Tullberg 1981; Nuyts and Michiels 1993).

Field-collected adults provided an opportunity to study mating behavior under conditions reflecting natural population dynamics, although the previous mating history was unknown. In many cricket species, female mating experience influences receptivity, mate choice, and copulation duration (Tinghitella 2014; Worthington and Kelly 2016). However, *U. meridionalis* exhibited extended mating events involving multiple spermatophore transfers despite the unknown reproductive history of the females. The frequent consumption of spermatophores suggests that these structures may function as post-nuptial gifts, potentially contributing to female nutritional intake and extended mating, although their physiological and fitness benefits remain to be investigated.
